# A prediction model for identifying high-risk lymph node metastasis in clinical low-risk papillary thyroid microcarcinoma

**DOI:** 10.1186/s12902-023-01521-0

**Published:** 2023-11-27

**Authors:** Hui Huang, Yunhe Liu, Song Ni, Shaoyan Liu

**Affiliations:** Department of Head and Neck Surgical Oncology, National Cancer Centre, National Clinical Research Centre for Cancer/Cancer Hospital, Chinese Academy of Medical Sciences and Peking Union Medical College, No. 17, Panjiayuan Nanli, Chaoyang District, Beijing, 100021 China

**Keywords:** Papillary thyroid microcarcinoma, High volume lymph node Metastasis, Extranodal extension, Risk factors, Nomogram

## Abstract

**Background:**

The presence of high-volume lymph node metastasis (LNM) and extranodal extension (ENE) greatly increases the risk of recurrence in patients with low-risk papillary thyroid microcarcinoma (PTMC). The goal of this research was to analyze the factors that contribute to high-risk lymph node metastasis in patients with low-risk PTMC.

**Methods:**

We analyzed the records of 7344 patients who were diagnosed with low-risk PTMC and treated at our center from January 2013 to June 2018.LNM with a high volume or ENE was classified as high-risk lymph node metastasis (hr-LNM). A logistic regression analysis was conducted to identify the risk factors associated with hr-LNM. A nomogram was created and verified using risk factors obtained from LASSO regression analysis, to predict the likelihood of hr-LNM.

**Results:**

The rate of hr-LNM was 6.5%. LASSO regression revealed six variables that independently contribute to hr-LNM: sex, age, tumor size, tumor location, Hashimoto’s thyroiditis (HT), and microscopic capsular invasion. A predictive nomogram was developed by integrating these risk factors, demonstrating its excellent performance. Upon analyzing the receiver operating characteristic (ROC) curve for predicting hr-LNM, it was observed that the area under the curve (AUC) had a value of 0.745 and 0.730 in the training and testing groups showed strong agreement, affirming great reliability.

**Conclusion:**

Sex, age, tumor size, tumor location, HT, and microscopic capsular invasion were determined to be key factors associated with hr-LNM in low-risk PTMC. Utilizing these factors, a nomogram was developed to evaluate the risk of hr-LNM in patients with low-risk PTMC.

## Background

Papillary thyroid microcarcinoma (PTMC) is defined as papillary thyroid carcinoma (PTC) with a diameter ≤ 1 cm [1]. Patients with PTMC generally have a favorable prognosis, with a disease-specific survival rate as high as 99% over a period of 10 years and a recurrence rate of 5% or less [2, 3]. The majority of diagnosed PTMCs are considered low-risk, characterized by intrathyroidal tumors without evidence of metastasis and adverse prognostic factors such as extrathyroidal extension (ETE) and no cytologic or molecular evidence (if performed) of aggressive disease [4]. According to the American Thyroid Association (ATA), active surveillance (AS) may be considered an alternative to immediate surgery [4]. Studies have demonstrated that AS can produce similar results to immediate surgery [5–7]. However, this approach is presently not extensively implemented in practice. Due to the lack of evidence, the potential risk of disease progression cannot be evaluated with certainty.

Lymph node metastasis (LNM) is one of the most important factors for PTC recurrence and survival. Studies have suggested a significant correlation between the number of metastatic lymph nodes (LNs) and recurrence. Sugitani et al. reported that patients with more than five metastatic LNs had a significantly higher recurrence rate (19% vs. 8%) [8]. Similarly, in a study by Leboulleux et al., patients who had more than 10 metastatic LNs (21%) or 6–10 metastatic LNs (7%) had significantly higher recurrence rates over a period of 10 years [9]. In the 2015 ATA guidelines, the presence of more than five metastatic LNs in pathologic N1 was identified as one of the criteria for upgrading low-risk to intermediate-risk. This increase in risk is associated with an increased risk of structural recurrence of approximately 15% [4]. Extranodal extension (ENE) has also been confirmed as a predictive factor for PTC recurrence and mortality [10–12].

Imaging methods like ultrasound and CT lack accuracy in assessing cervical LNM, particularly in the central region that is commonly affected in patients with clinically negative lymph node (cN0) PTC [13, 14]. Researched have revealed that the occult central lymph node metastasis (CLNM) rate in cN0 PTMC ranges from 15.3 to 60.9% [15–17]. The prevalence of high-volume lymph node metastasis (hv-LNM), which refers to the presence of more than five metastatic lymph nodes, in cN0 PTMC patients, has been documented to vary between 4% and 8.87%. and several risk factors have been established, including younger age, larger tumor size, male sex, BRAF V600E mutation, extrathyroidal extension, multifocality, capsular invasion, and microcalcification [18–22].

As previously stated, hv-LNM and ENE play a crucial role in the recurrence of PTMC patients. Hence, additional research is imperative to distinguish patients exhibiting these high-risk factors from the substantial pool of patients with low-risk PTMC. In this study, we defined LNM with any of these factors as high-risk LNM (hr-LNM). We conducted a retrospective analysis of the data of patients with low-risk PTMC. Our objective was to assist in clinical decision-making for managing low-risk PTMC by identifying the risk factors for hr-LNM.

## Materials and methods

### Patient selection and treatment

**Fig. 1:**
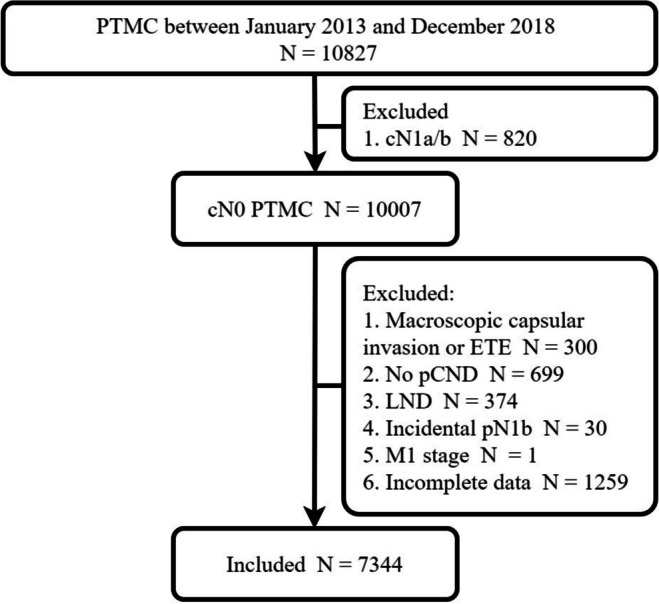
Illustration of the study design for the inclusion and exclusion criteria for patient enrollment

The medical records of 17,116 patients who underwent surgery for papillary thyroid carcinoma (PTC) at our center from January 2013 to December 2018 were retrospectively analyzed. Out of those, a total of 10,827 patients were diagnosed with Papillary Thyroid Carcinoma (PTC) where the tumor size did not exceed 10 mm on pathology assessment, also referred to as Papillary Thyroid Microcarcinoma (PTMC). Figure 1 displays the criteria for inclusion and exclusion. The clinical LN status was determined using preoperative ultrasound (US) results. The diagnosis of cN0 could only be confirmed if there were no signs of concern detected during the US examination, including abnormalities such as focal or diffuse hyperechogenicity, internal calcifications, cystic changes, or a round shape [23]. Tumors with macroscopic capsular invasion or extrathyroidal invasion, which were identified through US examination by observing penetration into the capsule, a discontinuous capsule, or invasion into perithyroidal tissues, were excluded. Thirty patients had LNM in the lateral neck based on postoperative pathological examination; however, they were negative on intraoperative frozen section examination and were excluded. We also excluded patients with incomplete data regarding tumor location and number of metastatic LNs. In the research study, a total of 3364 patients (45.8%) underwent preoperative fine-needle aspiration cytology (FNAC) examination, but the findings did not yield any details regarding variant types or differentiation. Finally, the analysis encompassed a total of 7344 patients.

Surgery included lobectomy or total thyroidectomy plus ipsilateral or bilateral prophylactic central neck dissection (CND) based on standard indications. Ipsilateral CND included prelaryngeal, pretracheal, and paratracheal LNs. Thyrotropin suppression therapy was administered postoperatively to achieve an appropriate level.

### Data collection

The collected data included sex, age, tumor size, tumor location, Hashimoto’s thyroiditis (HT), multifocality, bilaterality, microscopic capsular invasion, number of metastatic LNs, and ENE. The tumor size was recorded based on the largest dimension. Diagnosis of HT, multifocality, bilaterality, and microscopic capsular invasion was based on pathological results. Tumor location was recorded as upper, middle, lower, or multifocal based on intraoperative findings and pathological results. Staging was performed according to the American Joint Committee on Cancer TNM Stage for Thyroid Cancer (8th Edition, 2017) [24]. Initial risk stratification was performed according to the 2015 American Thyroid Association (ATA) guidelines [4].

### Statistical analysis

The main point of this study was hr-LNM, which was defined as LNM with more than five metastatic LNs or ENE. The collected *dataset* was randomly divided into training and validation cohorts at a ratio of 7:3, and the variables were compared. Categorical variables are presented as frequencies and proportions, whereas continuous variables are described as the means with standard deviations (SDs). In univariate analysis, the chi-square test or Fisher’s exact test was used to analyze categorical variables, while Student’s t test or rank-sum test was used to examine continuous variables. In the training cohort, LASSO logistic regression analysis was employed for multivariate analysis in order to identify independent risk factors and develop a predictive nomogram for hr-LNM. The accuracy of the nomogram was evaluated through the receiver operating characteristic (ROC) curve and calibration curve, with the range of the area under the receiver operating characteristic (ROC) curve (AUC) being 0.5 (indicating no discrimination) to 1 (indicating perfect discrimination). For evaluating the net benefit threshold of the prediction, decision curve analysis (DCA) was conducted. All statistical tests conducted were two-sided. A *P* value less than 0.05 was considered statistically significant, with R software (version 4.2.2) and MSTATA software being used for all statistical analyses.

## Results

### Clinicopathological characteristics

Table 1 presents the baseline characteristics of the study population. Of these, 2948 patients (40.1%) were classified as pN1a. Additionally, 269 patients (3.7%) had more than five metastatic LNs. ENE was observed in 264 (3.6%) patients. A total of 480 patients (6.5%) were identified to have hr-LNM. The most common histological variant was classic (81.9%, *n* = 6013), followed by follicular (15.2%, *n* = 1117), aggressive (0.3%, *n* = 24), and others (2.6%, *n* = 190). Aggressive variants included diffuse sclerosis (3), columnar cell (2), high cell (8), and solid variants (11).

**Table 1:** Patient demographics and baseline characteristics

Characteristic	Overall, *N* = 7,344^1^	Training Cohort, *N* = 5,141^1^	Internal Test Cohort, *N* = 2,203^1^	*P*-value^2^
**Sex**				0.633
** female**	5,617 (76%)	3,940 (77%)	1,677 (76%)	
** male**	1,727 (24%)	1,201 (23%)	526 (24%)	
**Age**				0.623
** Mean (SD)**	44 (10)	44 (10)	44 (10)	
**Tumor size**				0.477
** Mean (SD)**	0.64 (0.21)	0.64 (0.20)	0.63 (0.21)	
**Tumor location**				0.069
** upper**	1,019 (14%)	706 (14%)	313 (14%)	
** middle**	2,340 (32%)	1,617 (31%)	723 (33%)	
** lower**	1,090 (15%)	742 (14%)	348 (16%)	
** multifocal**	2,895 (39%)	2,076 (40%)	819 (37%)	
**HT**				0.502
** present**	1,756 (24%)	1,218 (24%)	538 (24%)	
** absent**	5,588 (76%)	3,923 (76%)	1,665 (76%)	
**Microscopic capsular invasion**				0.822
** absent**	3,889 (53%)	2,718 (53%)	1,171 (53%)	
** present**	3,455 (47%)	2,423 (47%)	1,032 (47%)	
**Bilaterality**				0.619
** absent**	5,633 (77%)	3,935 (77%)	1,698 (77%)	
** present**	1,711 (23%)	1,206 (23%)	505 (23%)	

^1^n (%)

^2^Pearson’s Chi-squared test; Welch Two Sample t-test

When examining the training cohort (*N* = 5141) and the internal test cohort (*N* = 2203), similar proportions of females and males were observed (*P* = 0.633). The mean age of patients in both cohorts was 44 ± 10 years. The mean tumor size for the training cohort was 0.64 ± 0.20 cm, and for the testing cohort, it was 0.63 ± 0.21 cm (*P* = 0.477). Furthermore, the distribution of tumor locations was comparable across the cohorts (*P* = 0.069). There were no significant differences between the cohorts in terms of the presence or absence of HT, microscopic capsular invasion, or bilaterality (*P* = 0.502, 0.822, and 0.619, respectively). Overall, the baseline characteristics of the study population were comparable across the training and internal test cohorts, indicating a representative sample for analysis.

### Univariate analyses of variables between patients with and without hr-LNM

In the training cohort, patients with hr-LNM were predominantly male and younger. Additionally, these patients had larger tumors and a significantly higher proportion of multifocal and bilateral tumors, as well as microscopic capsular invasion. In contrast, patients with hr-LNM had a lower proportion of coexisting HT. Similar differences were observed in the test cohort (See in Table 2).

**Table 2:** Comparison of variables between patients with and without high-risk LNM in the training cohort and internal test cohort

Characteristics	Training Cohort			Internal Test Cohort		
	**No high-risk LNM** *N* = 4,809^1^	**high-risk LNM** *N* = 332^1^	***P*-value** ^2^	**No high-risk LNM** *N* = 2,055^1^	**high-risk LNM** *N* = 148^1^	***P*-value** ^2^
**Sex**			< 0.001			< 0.001
** female**	3,733 (78%)	207 (62%)		1,588 (77%)	89 (60%)	
** male**	1,076 (22%)	125 (38%)		467 (23%)	59 (40%)	
**Age**			< 0.001			< 0.001
** Mean (SD)**	45 (10)	39 (10)		44 (10)	40 (9)	
**Tumor location**			< 0.001			0.003
** upper**	689 (14%)	17 (5.1%)		300 (15%)	13 (8.8%)	
** middle**	1,521 (32%)	96 (29%)		686 (33%)	37 (25%)	
** lower**	697 (14%)	45 (14%)		325 (16%)	23 (16%)	
**multifocal**	1,902 (40%)	174 (52%)		744 (36%)	75 (51%)	
**Tumor size**			< 0.001			< 0.001
** Mean (SD)**	0.63 (0.20)	0.73 (0.20)		0.63 (0.21)	0.73 (0.19)	
**HT**			0.006			0.003
** present**	1,160 (24%)	58 (17%)		517 (25%)	21 (14%)	
** absent**	3,649 (76%)	274 (83%)		1,538 (75%)	127 (86%)	
**Microscopic capsular invasion**			< 0.001			< 0.001
** absent**	2,582 (54%)	136 (41%)		1,113 (54%)	58 (39%)	
** present**	2,227 (46%)	196 (59%)		942 (46%)	90 (61%)	
**Bilaterality**			0.031			0.025
** absent**	3,697 (77%)	238 (72%)		1,595 (78%)	103 (70%)	
** present**	1,112 (23%)	94 (28%)		460 (22%)	45 (30%)	

### Multivariate analysis of risk factors for hr-LNM

**Fig. 2:**
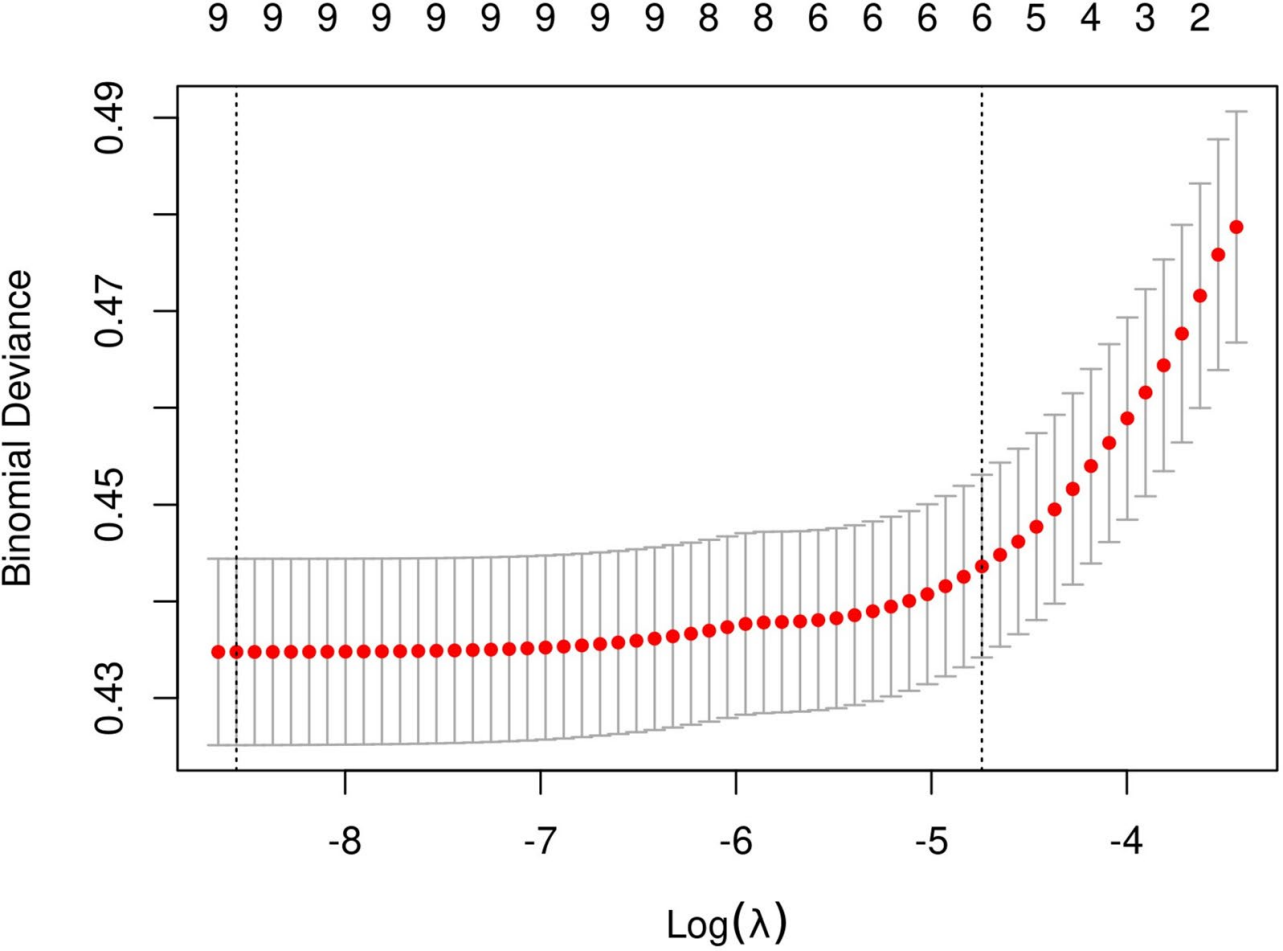
Lasso regression cross-validation plot

**Fig. 3:**
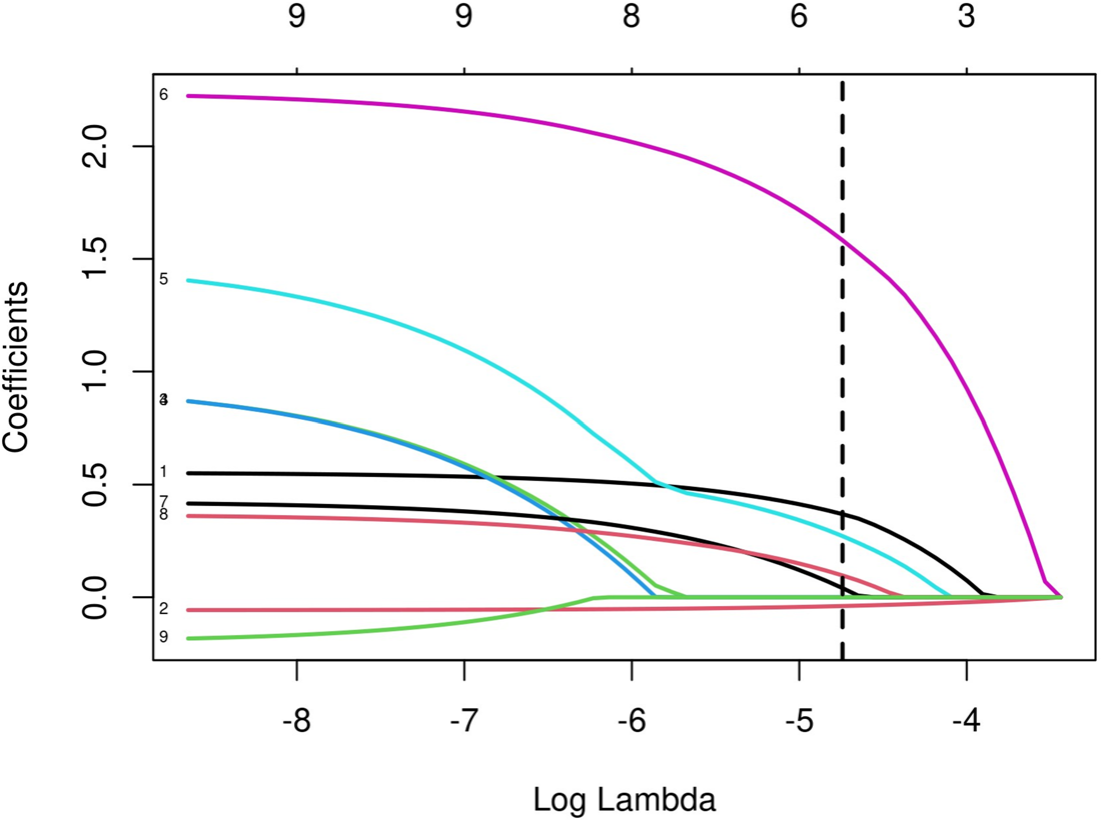
Lasso regression coefficient path plot

The initial model incorporated various potential predictors, such as sex, age, tumor location, tumor size, microscopic capsular invasion, bilateral tumors, and HT. LASSO regression analysis was used on the training cohort to identify significant variables. Figure 2 showed the Lasso Regression Cross-Validation Plot. The red dotted vertical line crosses over the optimal log λ, which corresponds to the minimum value for LASSO regression model. The two dotted lines represent one standard deviation from the minimum value. Figure 3 showed the LASSO coefficient profiles of the potential factors. Each curve represents a coefficient, and the x-axis represents the regularization penalty parameter. As λ changes, a coefficient that becomes non-zero enters the LASSO regression model. The resulting model consisted of six potential predictors: age, sex, tumor size, tumor location, HT, and microscopic capsular invasion. The investigation demonstrated that each of the six variables was capable of independently predicting the presence of hr-LNM according to Table 3. The likelihood of hr-LNM was noticeably greater in male patients compared to female patients (OR = 1.74, 95% CI 0.93–0.96; *P* < 0.001). The older patients demonstrated a decreased incidence of high-risk lymph node metastasis compared to the younger patients (OR = 0.94, 95% CI 0.93–0.96; *P* < 0.001). Tumor size also plays a significant role in predicting hr**-**LNM. The probability of hr**-**LNM significantly increased with tumor size (OR = 9.21, 95% CI 5.10-16.76; *P* < 0.001). Furthermore, patients with microscopic capsular invasion hr**-**LNM (OR = 1.44, 95% CI 1.13–1.83; *P* = 0.003). In contrast, patients without coexisting HT had a significantly higher risk of hr**-**LNM than those with HT (OR = 1.54, 95% CI 1.14–2.11; *P* = 0.006). Moreover, the risk of hr**-**LNM varied significantly depending on tumor location, with the highest risk observed in patients with multifocal tumors (OR = 3.97, 95% CI 2.45–6.88; *P* < 0.001). The test group also exhibited comparable findings.

**Table 3:** Results of multivariate logistic regression for training cohort and internal test cohort

Characteristic		Training Cohort			Internal Test Cohort		
		**OR** ^1^	**95% CI** ^1^	***P*-value**	**OR** ^1^	**95% CI** ^1^	***P*-value**
Sex	**female**	-	-		-	-	
	**male**	1.74	1.36, 2.23	< 0.001	1.93	1.34, 2.77	< 0.001
Age		0.94	0.93, 0.96	< 0.001	0.96	0.94, 0.98	< 0.001
Tumor location	**upper**	-	-		-	-	
	**middle**	2.58	1.56, 4.53	< 0.001	1.14	0.60, 2.28	0.702
	**lower**	2.59	1.48, 4.73	0.001	1.55	0.77, 3.26	0.226
	**multifocal**	3.97	2.45, 6.88	< 0.001	2.24	1.24, 4.34	0.011
Tumor size		9.21	5.10, 16.76	< 0.001	9.06	3.79, 21.98	< 0.001
HT	**present**	-	-		-	-	
	**absent**	1.54	1.14, 2.11	0.006	2.12	1.32, 3.56	0.003
Microscopic capsular invasion	**absent**	-	-		-	-	
	**present**	1.44	1.13, 1.83	0.003	1.50	1.05, 2.16	0.027

^1^ *OR *Odds Ratio, *CI *Confidence Interval

### Development of a prediction model for predicting hr-LNM

**Fig. 4:**
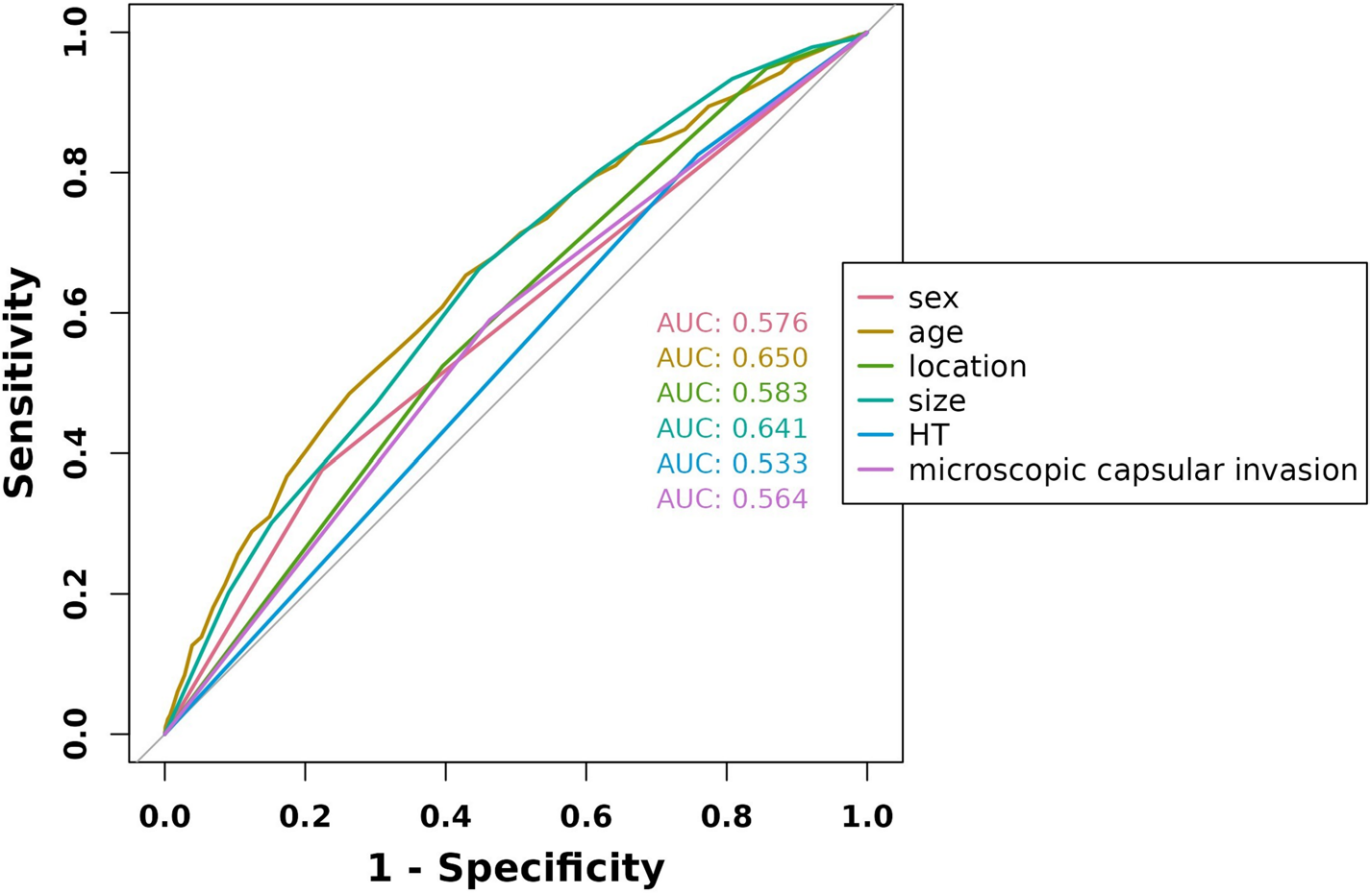
ROC curve analysis of six candidate predictors

**Fig. 5:**
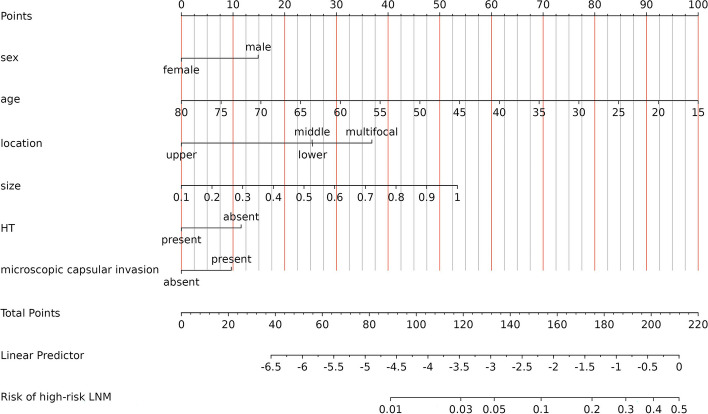
Nomogram prediction model of predicting hr-LNM

**Fig. 6:**
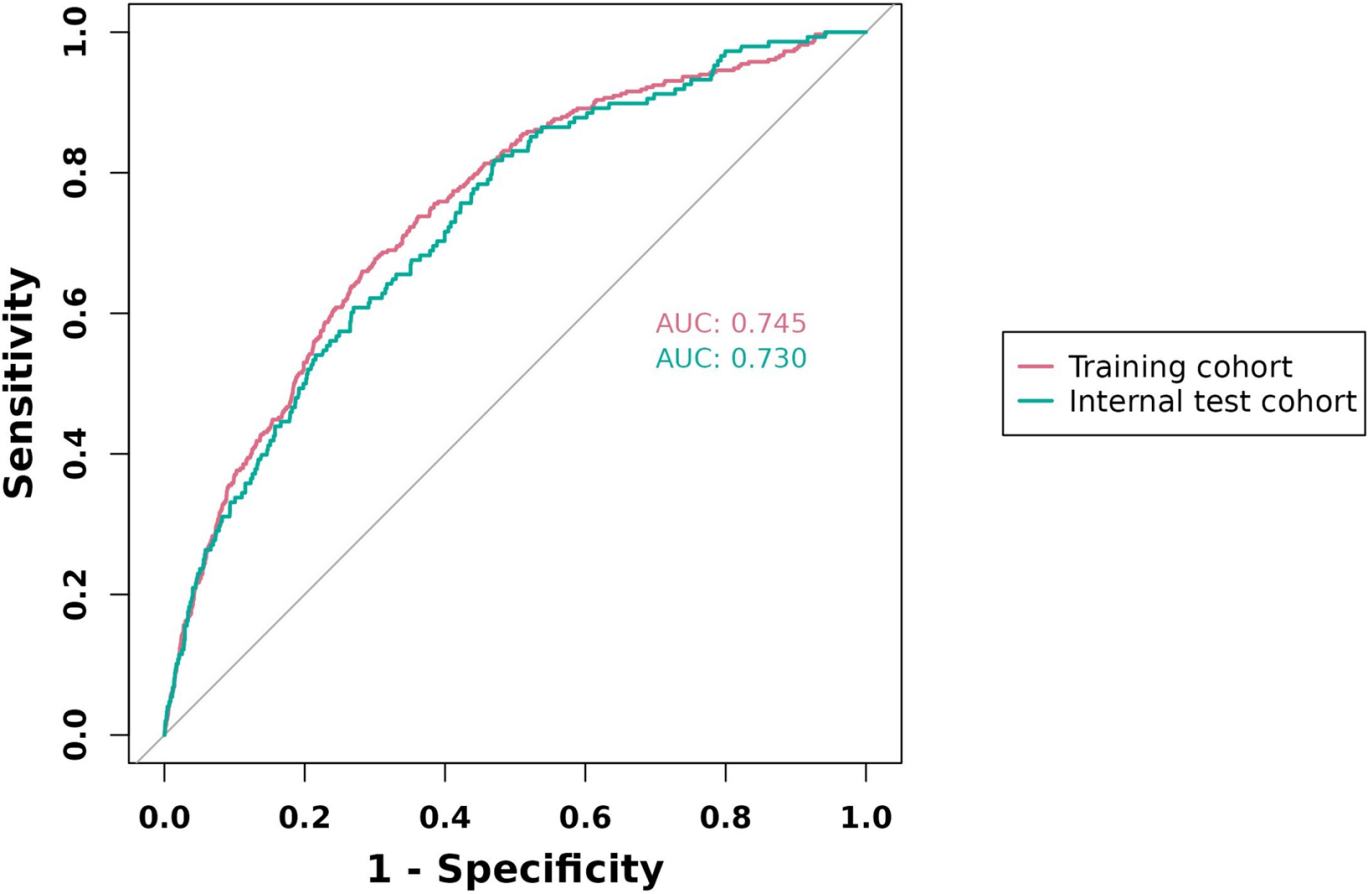
ROC curves of the nomogram prediction model

**Fig. 7:**
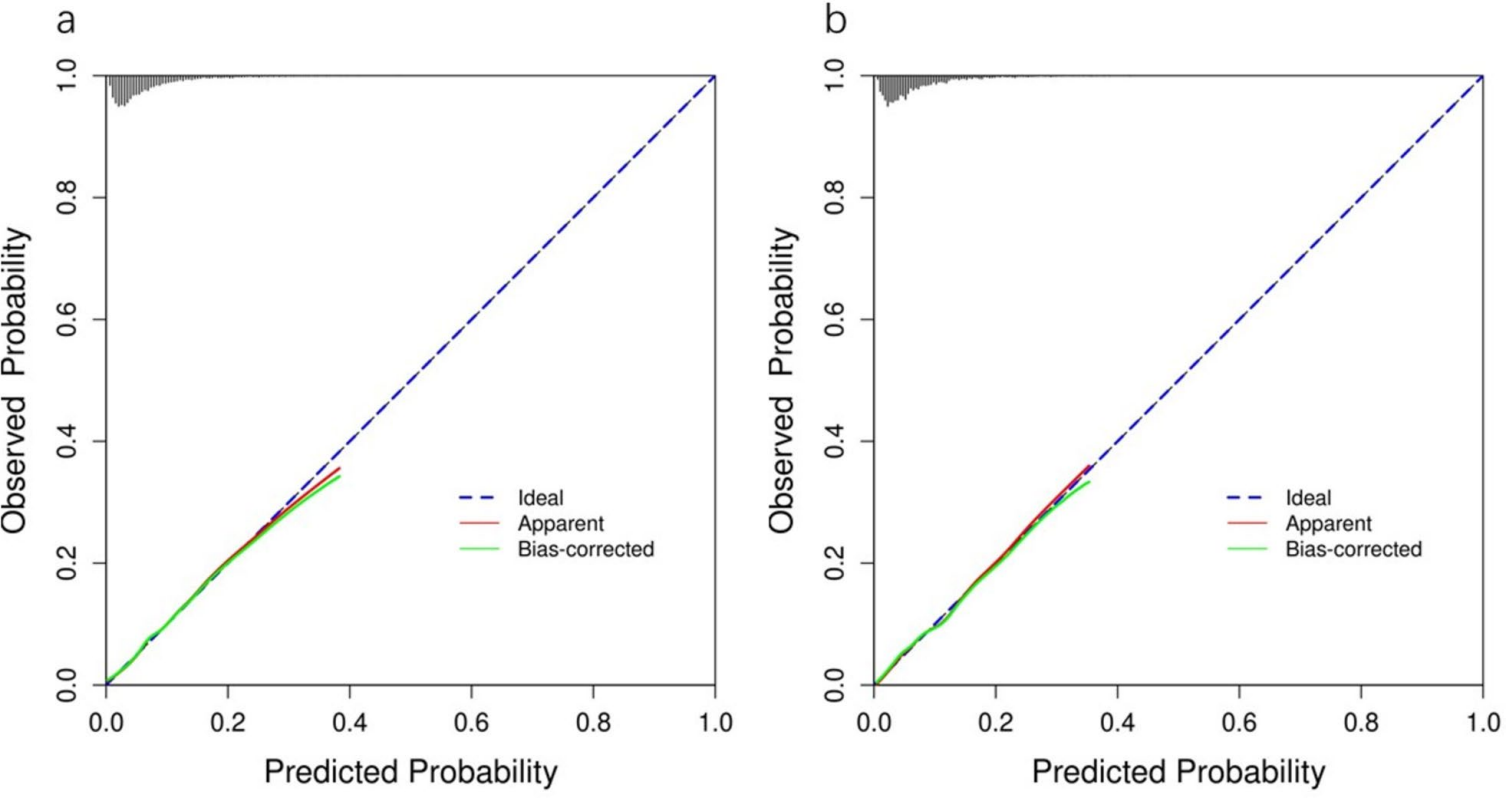
Calibration curve of the nomogram prediction model for the training cohort (**a**) and the internal test cohort(**b**)

**Fig. 8:**
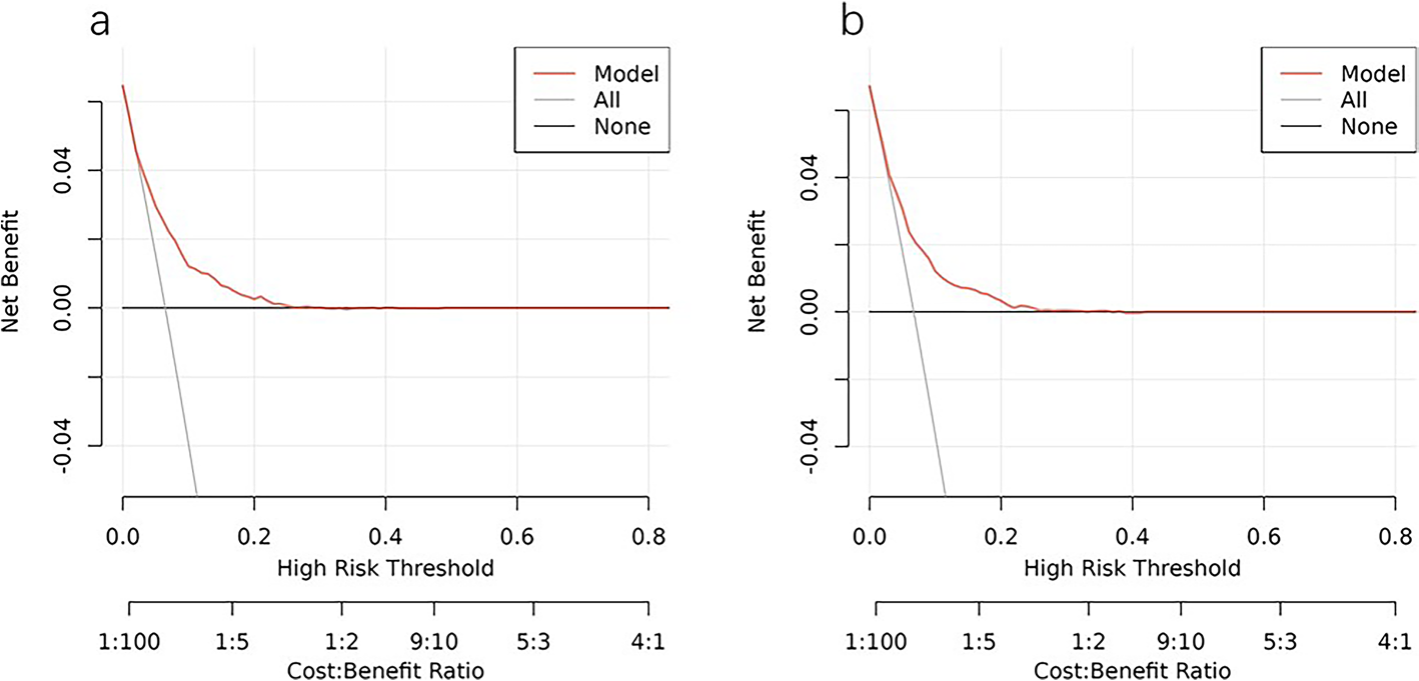
Decision curve analysis of the nomogram of the training cohort (**a**) and the internal test cohort(**b**)

The ROC analysis of the six aforementioned variables resulted in AUC values greater than 0.5 (Fig. 4). As a result, these variables were incorporated into the development of a nomogram (Fig. 5). The area under the curve (AUC) measurements of this model for the training and testing groups were 0. 745 and 0. 730, respectively (Fig. 6) The nomogram underwent 1,000 bootstrap analyses to internally validate and calibrate its accuracy. Calibration plots in Fig. 7 exhibit a robust correlation between predicted and observed hr-LNM across various cohorts. By closely resembling the ideal curve in the calibration curve, these findings suggest that the original nomogram remained effective and accurately predicted outcomes compared to the actual results when applied to the validation sets. Figure 8a and b show the DCA curves related to the nomogram. A high-risk threshold probability indicates the likelihood of significant discrepancies in the model’s predictions when clinicians encounter major flaws while using the nomogram. This study underscores the significant advantages that the nomogram brings to clinical applications.

## Discussion

Active surveillance (AS) has been proven to have favorable clinical outcomes similar to those achieved with immediate surgery for low-risk PTMC [5–7], and the American Thyroid Association (ATA) guidelines endorse active surveillance (AS) as a viable option for low-risk PTMC [4].

Various research studies have documented that the prevalence of occult central lymph node metastasis (CLNM) in individuals with cN0 PTMC varies considerably, ranging from15.3–60.9% [14–16]. While certain researches have implied that occult CLNM does not have a substantial effect on the overall prognosis of patients with PTC [12, 25], an elevated number of metastatic lymph nodes would heighten the likelihood of disease recurrence. Sugitani et al. reported that patients with more than five metastatic LNs had a significantly higher risk of recurrence (19% vs. 8%) [9]. Similarly, in a study conducted by Leboulleux et al., more than 10 metastatic LNs (21%) or 6–10 metastatic LNs (7%) were associated with a significantly higher recurrence rate [9]. In the 2015 ATA guidelines, the presence of pathologic N1 with more than 5 metastatic LNs was identified as one of the criteria for upgrading low-risk PTC to intermediate-risk PTC, which is associated with an approximately 15% increase in the risk of structural recurrence [4]. Additionally, there may be a higher recurrence of lymph nodes reappearing in individuals with AS compared to those who undergo immediate surgery [26]. Therefore, in our view, patients who are at a high risk of having hr-LNM should contemplate undergoing prompt surgical treatment.

Multiple research studies have provided evidence that various clinical and pathological characteristics significantly contribute to the risk of occult CLNM in cN0 PTMC, including male sex, younger age, large tumor size, multifocality, extrathyroidal extension, and lymphovascular invasion [15, 27]. Studies have also investigated the predictive factors for hv-LNM. In a retrospective study, 13.4% of the patients with PTC had hv-CLNM. The results further indicated that factors such as age, BRAF V600E mutation, tumor size, and calcification independently influence the likelihood of hv-CLNM [22]. Similarly, another study investigated patients with PTMC and found a 6.4% incidence rate of hv-CLNM. Preoperative ultrasonic features, including microcalcifications, larger tumor size (> 7 mm), and capsule invasion, were established as independent predictors [19]. These findings were consistent with those of a meta-analysis, which concluded that younger age, tumor size > 5 mm, male sex, extrathyroidal extension, multifocality, microcalcification, capsular invasion, and rich blood flow were risk factors for hv-CLNM [21]. These studies included pathological features such as extrathyroidal extension, which cannot be assessed before surgery. Moreover, the analysis of the BRAF V600E mutation through gene testing has not been widely conducted in the context of fine-needle aspiration (FNA). Therefore, there is a need to conduct research on predictive factors and develop a simplified prediction model to aid in decision-making for low-risk PTMC.

ENE has also been widely recognized as a significant risk factor for recurrence [10, 11] and cancer-related mortality [12] of PTC. Hence, we consider it crucial to conduct evaluations of ENE in occult CLNM, despite the incidence may be low, as was observed in only 3.6% of the patients in our study.

Hv-LNM and ENE were considered high-risk features for LNM in the present study. LNM with either of these features was defined as high-risk LNM (hr-LNM). Multivariate analysis revealed that sex, age, tumor location, tumor size, microscopic capsular invasion, and HT were independent risk factors for hr-LNM. Based on the findings, we created a model that accurately forecasts the probability of hr-LNM in patients with clinical low-risk PTMC.

In our model, we consider age and size as continuous variables instead of relying on predetermined cutoff values mentioned in earlier studies [21, 22, 28]. We have confidence in the potential of this innovative approach to enhance the precision of the forecasting model. The identification of HT as a protective factor for PTC [29] is based on its correlation with a decreased occurrence of extrathyroidal extension and a lower frequency of lymph node metastasis. Based on our previous investigation, individuals diagnosed with PTMC and coexisting HT presented a reduced incidence of LNM, a decreased quantity of metastatic lymph nodes (LNs), and a lower incidence of ENE compared to those without HT [30]. Additionally, for the first time, our model incorporates tumor location as a factor during its development. Our findings revealed a remarkable increase in the risk of hr-LNM in tumors situated in the middle and lower regions of the thyroid compared to those located in the upper part. Based on prior knowledge that tumors located in the middle and lower thyroid have a higher rate of occult CLNM, this finding aligns with previous studies [31]. Furthermore, patients with multifocal tumors have a significantly increased likelihood of hr-LNM. In our model, we accounted for both macroscopic and microscopic multifocality. We believe that utilizing the detection of macroscopic multifocality through preoperative ultrasound could greatly enhance the accuracy of predicting hr-LNM.

Microcarcinomas located near the inner or upper side of the thyroid gland are not suitable candidates for active surveillance [5]. Patients with tumors showing capsule involvement or extrathyroid extension found through preoperative US examination were not included. In our group of patients, we lacked information on the distance between the tumor and thyroid capsule. Consequently, we incorporated microscopic capsular invasion into the prediction model, although its relevance for guiding treatment decisions may be limited. Speculating on the risk of hr-LNM, it could potentially be elevated in cases where a tumor is found adjacent to the thyroid capsule rather than within the thyroid. Further research is necessary to explore the correlation between the tumor’s distance to the capsule and the risk of hr-LNM, thereby enhancing the accuracy of prediction models.

Our study has some limitations. As this study was conducted retrospectively, the absence of predesigned criteria and the variable sample size may lead to the introduction of biases. The majority of patients did not go through genetic testing. Hence, no record of the BRAFV600E mutation was documented. FNAC results regarding differentiation and variants were not obtained prior to surgery. Before surgery, it is common for the majority of patients to not receive any diagnostic information about differentiation, variants, or the presence of BRAF V600E. Therefore, our inclusion criteria for clinical low-risk PTMC were cN0 and no macroscopic ETE. Twenty-four patients were found to have aggressive variants according to the pathological findings. Nonetheless, we are confident that the clinical significance of our findings in assisting treatment choices will not be affected. Further analysis should be conducted to validate this model by undertaking a meticulously planned prospective study.

## Conclusion

This study developed a reliable prediction model to assess the risk of hr-LNM in clinical low-risk PTMC patients, which can substantially aid clinicians in identifying those who would benefit from surgery, and ultimately improve patient outcomes.

## Data Availability

The authors of this article are dedicated to providing unrestricted access to the raw data that supports the presented conclusions. The raw materials and data will be made available without any reservations or restrictions. Hui Huang (E-mail: huanghuinj@163.com) is responsible for providing the raw materials and data.

## Abbreviations

PTC: Papillary thyroid carcinoma
PTMC: Papillary thyroid microcarcinoma
LNM: Lymph node metastasis
CLNM: Central lymph node metastasis
HT: Hashimoto's thyroiditis
ETE: Extrathyroidal extension
ENE: Extranodal extension
CND: Central neck disssetion
AS: Active surveillance
